# Janus kinase and signal transducer and activator of transcription inhibitors in type 1 diabetes and immune checkpoint–related diabetes: current status and future perspectives

**DOI:** 10.3389/fimmu.2025.1571247

**Published:** 2025-06-04

**Authors:** Bowei Su, Zhi-Lin Luan, Haixia Liu, Jaakko Tuomilehto, Xiaochen Ji

**Affiliations:** ^1^ Department of Endocrinology and Metabolism, the Second Affiliated Hospital of Dalian Medical University, Dalian, China; ^2^ Advanced Institute for Medical Sciences, Dalian Medical University, Dalian, China; ^3^ Department of Public Health, University of Helsinki, Helsinki, Finland

**Keywords:** type 1 diabetes, JAK/STAT signaling pathway, JAK inhibitors, immune checkpoint inhibitors, immune checkpoint inhibitor-induced diabetes, immunotherapy

## Abstract

Type 1 diabetes (T1D) is an autoimmune-mediated disorder that leads to the destruction of pancreatic beta-cells, insulin deficiency, and chronic hyperglycemia. It is one of the most common childhood endocrine disorders. Recent evidence indicates that aberrant Janus kinase–signal transducer and activator of transcription (JAK/STAT) signaling exacerbates T1D by promoting the production of proinflammatory cytokines and chemokines. By blocking JAK-mediated phosphorylation of STAT proteins, JAK inhibitors help alleviate cytokine-driven inflammation, reduce insulin requirements, and relieve complications such as painful peripheral neuropathy, potentially preserving residual beta-cell function and improving glycemic control. Moreover, emerging data underscore the potential synergy between JAK inhibitors and immune checkpoint therapies targeting the programmed cell death protein 1 (PD-1) pathway, as PD-1/Programmed cell death ligand 1 (PD-L1) inhibitors used in antitumor therapy can induce immune checkpoint inhibitor–induced diabetes (CPI-DM). This review examines the impact of JAK inhibitors on beta-cells and immune cells in T1D, along with their safety profiles and adverse effects. It explores the potential benefits and risks of combining JAK inhibitors in the management of CPI-DM associated with anti–PD-1/PD-L1 therapy. In conclusion, while JAK inhibitors have demonstrated the potential to reduce inflammation and preserve beta-cell function in preclinical studies, further clinical trials are needed to confirm their long-term safety and efficacy in patients with T1D and CPI-DM.

## 1 Introduction

Type 1 diabetes (T1D) is an autoimmune disease characterized by the immune system’s aberrant recognition and subsequent destruction of pancreatic beta-cells (1). Similarly, immune checkpoint inhibitor-associated diabetes (CPI-DM), while triggered by the administration of immune checkpoint inhibitors, results from an overactive immune response that ultimately leads to beta-cell destruction (2). Both conditions share a common immune-mediated mechanism of beta-cell injury. In both T1D and immune checkpoint inhibitor-associated diabetes, abnormal activation and infiltration of T cells and other immune cells are observed, accompanied by a significant release of inflammatory cytokines, including interferons (IFNs) and interleukins, which amplify the inflammatory process within the pancreas.

The Janus kinase-signal transducer and activator of transcription (JAK/STAT) pathway plays a central role in cytokine signaling by regulating a variety of cytokines critical for modulating immune and inflammatory responses (3). Given that the JAK/STAT pathway is instrumental in the expression and activation of multiple inflammatory and immunoregulatory mediators, its dysregulation may facilitate the immune-mediated destruction of pancreatic beta-cells. Indeed, studies have shown that inhibition of the JAK pathway holds promise in mitigating excessive immune responses and protecting beta-cells from damage (4, 5).

Accordingly, this review summarizes and discusses the latest clinical research on the JAK/STAT pathway and its associated pharmacological agents in the treatment of T1D, including an evaluation of their potential side effects. Additionally, we examine the impact of JAK inhibitors on the programmed cell death protein 1/programmed cell death ligand 1 (PD-1/PD-L1) pathway and explore their prospective applications in managing immune checkpoint inhibitor-associated diabetes.

## 2 The role and mechanism of the JAK/STAT signaling pathway in type 1 diabetes

It is commonly recognized that a hallmark of T1D is the elevated expression of human leukocyte antigen (HLA-I) molecules on pancreatic beta-cells (6–8). This upregulation of HLA proteins is thought to be associated with aberrant processing and presentation of antigens to activate autoreactive MHC class I-restricted CD8 T cells, which ultimately causes beta-cell destruction, contributing to the pathogenesis of T1D (8–11). The persistent autoimmune response triggers kinase and transcription factor activation, creating a vicious inflammatory cycle. Within this process, the JAK/STAT signaling pathway serves as a central mechanism driving inflammatory cascade amplification by directly regulating the expression of pro-inflammatory genes. Ongoing autoimmune activity and the activation of kinases and transcription factors lead to additional inflammatory responses, with the JAK/STAT signaling pathway being a major contributor (12, 13).

**Figure 1:**
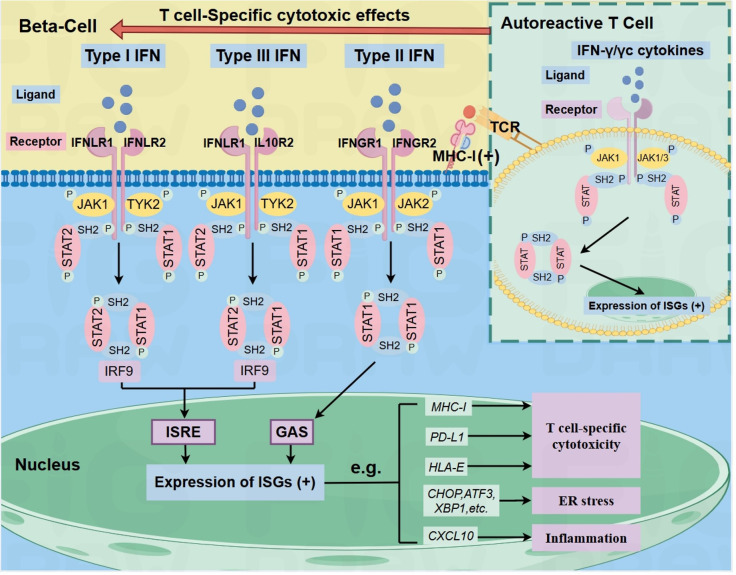
IFN signaling in beta-cells and autoreactive T-cells (inset) via JAK/STAT pathway in T1D. The binding of type I, II, or III IFNs to their respective receptors activates distinct JAK proteins, leading to STAT phosphorylation and, ultimately, the upregulation of ISGs, such as ER stress markers (CHOP, ATF3, XBP1), and CXCL10, which activate ER stress and inflammation in beta-cells. It also enhances beta-cell surface MHC-I, PD- L1, and HLA-E, enabling autoreactive CD8+ T cell recognition. In autoreactive T cells, IFN-y or common y-chain cytokines activate the JAK/STAT signaling pathway, leading to the upregulation of pro-inflammatory genes. This amplifies T cell-mediatedinflammation and may enhance T cell activation, thereby exacerbating autoimmune damage and contributing to the development of TID. Arrows indicate pathway directionality only. Black (+) symbols indicate transcriptional upregulation. ISGS: IFN-stimulated genes.

The JAK family—comprising JAK1, JAK2, JAK3, and TYK2—consists of non-receptor tyrosine kinases that are essential for cytokine signaling. These kinases mediate immune responses and the signaling of hormones, growth factors, and interferons (IFNs) (14) (see **Figure 1**). IFNs are classified into three types, each activating distinct receptor-kinase complexes and downstream pathways: Type I IFNs (e.g., IFNα/β) bind IFNAR1/IFNAR2 receptors, activating JAK1 and TYK2 to phosphorylate STAT1-STAT2 heterodimers. These heterodimers complex with IRF9 (forming ISGF3) and drive antiviral responses via interferon-sensitive response elements (ISRE). Type II IFN (IFN-γ), signaling through IFNGR1/IFNGR2 receptor complex, which recruits JAK1 and JAK2. This pathway leads to the formation of STAT1 homodimers that bind to gamma-activated sequences (GAS) in the promoters of pro-inflammatory genes. Type III IFNs (e.g., IFN-λ) act through the IFNLR1/IL10R2 receptor complex, similarly engaging JAK1 and TYK2 and triggering the formation of STAT1-STAT2-IRF9 complexes, thereby promoting antiviral gene expression via ISREs. Following ligand binding, all three IFN receptor type undergo conformational changes and dimerization, enabling JAK trans-phosphorylation. This process initiates the phosphorylation and release of STAT proteins, which dimerize and translocate into the nucleus to regulate gene expression. In T1D, Russell et al. emphasize that Type I/III IFNs (typically involved in antiviral responses) and Type II IFN (produced by autoreactive immune cells) synergistically activate the JAK/STAT pathway. This amplifies beta-cell inflammation and contributes to increased HLA-I expression, further exacerbating autoimmune-mediated beta-cell destruction (6).

In the early stage of T1D, TYK2—a candidate gene in T1D—plays a critical role in beta cells following viral infection (e.g., via polyinosinic-polycytidylic acid, a viral RNA mimic) or cytokine stimulation (e.g., IFN-α), where it mediates IFN-I signaling (7, 8). Upon IFN-α stimulation, the activation of the TYK2-STAT1/STAT2-IRF9 axis ultimately leads to enhanced expression of apoptosis- and inflammation-related genes in pancreatic beta-cells, including HLA-class I proteins, C-X-C motif chemokine ligand 10 (CXCL10), and endoplasmic reticulum (ER) stress markers (7, 9, 10). Recent studies have revealed that pancreatic beta-cells upregulate both membrane-bound and soluble forms of HLA-I under IFN stimulation, with the latter potentially influencing the islet inflammatory process during autoimmune attack by modulating the activation status of immune cells (11). It has been shown that TYK2 knockout in human stem cell–derived islets reduces their susceptibility to T cell–mediated cytotoxicity (12). Thus, TYK2 inhibition has emerged as a promising therapeutic strategy for preventing or treating T1D.

Currently, the molecular mechanisms governing JAK/STAT signaling in beta-cells during autoimmune attack remain incompletely clucidated, and targeted therapies against the JAK/STAT pathway still face certain limitations. Recent findings indicate that signal-regulatory protein alpha (SIRPα) can regulate the activity of membrane-associated HDAC6 in T1D cells, thereby modulating STAT1 phosphorylation and activation (13). Loss of SIRPα has been observed in the beta-cells of people with newly diagnosed T1D, resulting in a reduced ability of STAT1 to respond transcriptionally to pro-inflammatory cytokines, including IFN-γ. Beyond the primary mechanism of beta-cell survival via phosphorylation, cytokine-induced STAT1 acetylation may also contribute to the regulation of beta-cell survival (13). Studies have shown that STAT6 maintains beta-cell survival by activating anti-apoptotic genes such as MCL1, BCL2L1, and SIRPα. However, in pancreatic islets of individuals with T1D, STAT6 expression is significantly reduced, leading to impaired protective function for beta-cell. Leslie et el found that reduced STAT6 activity correlates with decreased SIRPα expression, and genetic ablation of SIRPα can directly compromises the cytoprotective effects mediated by IL-4/IL-13 (14). Therefore, while JAK inhibitors suppress pathogenic IFN signaling, they may concurrently block the IL-4/IL-13-STAT6-SIRPα protective axis, thereby limiting therapeutic efficacy. This dual inhibitory effect on both pathogenic and protective signaling pathways underscores the necessity and challenges of achieving precise modulation of the JAK/STAT network in T1D therapeutics.

## 3 Current potential JAK/STAT-related drugs for T1D

At present, several JAK/STAT-related immunomodulators have demonstrated efficacy in both clinical and animal studies for T1D (5, 15–17). These findings indicate that pharmacological modulation of the JAK/STAT pathway represents a potential therapeutic and preventive strategy for T1D (18, 19). Notably, some researchers propose that the therapeutic efficacy of JAK inhibitors may represent a class effect, suggesting that other JAK inhibitors (e.g., tofacitinib) may exhibit similar therapeutic potential (5). Therefore,through a search of Pubmed, Cochrane Library, Web of Science, and ClinicalTrials.gov, we summarized the current JAK/STAT-related drugs and Chinese herbal ingredients that may affect the mechanisms of diabetes and their related mechanisms, including potential adverse effects (see **Table 1**).

**Table 1:** Overview of JAK/STAT pathway inhibitors and their effects in T1D treatment.

Category	Agent	Experimental model/subjects	type	Target	Function	Common adverse event
Western Medicine	Baricitinib	EndoC-βH1 β-cells	Pre-clinical Trial	JAK1,JAK2	Protect β-cells against deleterious effects of proinflammatory cytokines	Hypoglycaemia
		T1DM and T2DM patients	Clinical Trial		Inhibit inflammation and preserve β-cell function	
	Tofacitinib	RA patients with T2DM	Clinical Trial	JAK1,JAK2 and JAK3 (mainly)	Mitigate insulin resistance and hyperglycemia	Hypoglycaemia
		STZ-induced Wistar rat model	Pre-clinical Trial			
	Ruxolitinib	Ifnar1−/− LEW.1WR1 rats	Pre-clinical Trial	JAK1,JAK2	Prevention of insulitis and inflammatory cell infiltration	Not described
		A patient with STAT1 gain-of-function disease	Case Report		Mitigate insulin resistance and hyperglycemia	
	Liraglutide	Jurkat E6-1 cells	Pre-clinical Trial	JAK2-STAT4	Inhibit Th1 cell differentiation and production of interferon γ	Gastrointestinal symptoms(nausea);Hypoglycemia and hyperglycemia with ketosis
		Ifnar1−/− LEW.1WR1 rats	Pre-clinical Trial	JAK-STAT pathway	Significant weight loss and adjunctive insulin-modulating effects	
		HFD-induced diabetes C57/BL6 mouse model	Pre-clinical Trial	JAK-STAT-SIRT1 pathway	Increase islet β cell number and function	
		T1DM and T2DM patients	Clinical Trial		Improves glycaemic control	
	Deucravacitinib/BMS-986165	RIP-LCMV-GP mice	Pre-clinical Trial	TYK2	reduce systemic and tissue-localized inflammation, prevent β cell death, and delay T1D onset	Not described
		NOD mouse model	Pre-clinical Trial			
		the human EndoC-βH1 β-cell line	Pre-clinical Trial		Protect β-cells from proinflammatory cytokine damage	
	BMS-986202	NOD mouse model	Pre-clinical Trial	TYK2	Reduce systemic and tissue-localized inflammation, prevent β cell death, and delay T1D onset	Not described
	LN3103801	NOD mouse model	Pre-clinical Trial	TYK2	Prevent and reverse anti‐PD‐L1‐induced diabetes by blocking IFNγ and γc cytokine activities	Not described
	ABT 317	NOD mouse model	Pre-clinical Trial	TYK2	Block the effect of cytokines by inhibiting MHC class I upregulation	Not described
	AZD1480	NOD mouse model	Pre-clinical Trial	TYK2	Block the effect of cytokines by inhibiting MHC class I upregulation	Not described
	TYK2iA/TYK2iB	the human EndoC-βH1 cells and islets	Pre-clinical Trial	TYK2	Protect β-cells against deleterious effects of proinflammatory cytokines	Not described
	Tyrphostin AG490	NOD mouse model	Pre-clinical Trial	JAK2 and JAK3	Mitigate hyperglycemia	Not described
		STZ-induced BLAB/c mice model				Not described
Herbal Ingredients	Punicalagin	STZ-induced Wistar rat model		IL6-JAK-STAT3 pathway	Reduces inflammation and oxidative stress, preventing pancreatic damage and insulitis	Not described
		Rat pancreatic β-cells				
	Asaronic acid	Mouse macrophage-like cell line J774A.1		IL6-JAK2-STAT1/2 pathway	Inhibit inflammation	Not described

### 3.1 Baricitinib

Baricitinib is an orally administered, reversible, immunosuppressant that competitively targets the JAK family, predominantly acting on the JAK1 and JAK2 subtypes. By inhibiting the phosphorylation of different STAT proteins (e.g., STAT1, STAT3), baricitinib disrupts downstream signaling of specific cytokines (e.g., IFN-γ, IL-6, IFN-α). It does so by competitively binding to the ATP pocket of JAK, thereby reducing the expression of inflammatory genes. Baricitinib is a selective and reversible JAK inhibitor with a high affinity for JAK1 and JAK2 and a significantly weaker inhibition of JAK3 (20). Currently, it has been approved for multiple inflammatory and autoimmune diseases, including alopecia areata (21), graft-versus-host disease (22, 23) and Corona Virus Disease 2019 (24). These approvals have been supported bypromising clinical outcomes, and a favorable safety profile demonstrated in treatment evaluations (25).

*In vitro* studies using IFN-α-stimulated human beta-cells have demonstrated that baricitinib exerts multifaceted protective effects by suppressing the overexpression of MHC class I molecules, alleviating ER stress, reducing the secretion of pro-inflammatory chemokines, and inhibiting apoptotic pathways (9, 26). These mechanistic insights further elucidate the drug’s direct cytoprotective actions in beta cells.

The BANDIT trial, a multicenter, double-blind, placebo-controlled study conducted in Australia, tested whether baricitinib could slow the immune-mediated destruction of pancreatic beta-cells in individuals aged 10–30 years with newly diagnosed T1D (5).Participants were randomized in a 2:1 ratio to receive baricitinib 4 mg/day or placebo for 48 weeks. This was followed by an additional 48-week observation period after drug cessation. Results showed that beta-cell function in the baricitinib group was significantly higher than in the control group after 48 weeks of treatment, as evidenced by mixed-meal–stimulated C-peptide measurements. Beta-cell function was assessed through standardized mixed-meal tolerance tests measuring blood C-peptide levels, with the baricitinib group demonstrating a median stimulated mean C-peptide level of 0.65 nmol/L/min (IQR 0.31-0.82) versus 0.43 nmol/L/min (IQR 0.13-0.63) in placebo. This quantitative evaluation of endogenous insulin production confirmed superior beta-cell preservation with baricitinib.

The authors found that baricitinib exhibited a favorable short-term (48-week) safety profile, with its partial JAK inhibitory characteristics potentially reducing long-term immunosuppressive risks. This pharmacological feature distinguishes it from traditional potent immunosuppressants (e.g., anti-thymocyte globulin) and offers a safer strategy for sustained immune modulation in T1D. Given the irreversible beta-cell damage observed in most patients at clinical T1D diagnosis, the study has redefined the therapeutic goal as “preservation of beta-cell function” rather than “complete insulin discontinuation,” driving a paradigm shift from *“cure-oriented”* to *“functional preservation-oriented”* approaches. This shift aligns with the current pathophysiological reality of T1D and provides a strategic reference for future applications of JAK inhibitors in T1D management (5).

### 3.2 Tofacitinib

Tofacitinib is another potent and selective inhibitor of the JAK family. Unlike baricitinib, it preferentially inhibits JAK1 and JAK3, with some inhibitory effects also on JAK2. By reducing signal transduction by various cytokines such as IL-2, IL-4, IL-6, and IFN-γ, tofacitinib decreases T-cell and B-cell activation. Tofacitinib has been approved by the U.S. Food and Drug Administration (FDA) for the treatment of moderate-to-severe RA (27, 28), psoriatic arthritis (29), and ulcerative colitis (30–33), among other inflammatory and autoimmune diseases (34, 35).

A study employing a double-transgenic mouse model with pancreatic beta-cell-specific expression of inhibiting interleukin-15(IL-15) and its receptor subunit, interleukin-15 receptor alpha (IL-15Rα), demonstrated that Tofacitinib-mediated suppression of IL-15 signaling not only reversed hyperglycemia but also eliminated mononuclear cell infiltration in the islets of IL-15/IL-15Rα double-transgenic mice (36). Furthermore, inhibition of IL-15 signaling during the prediabetic phase in NOD mice delayed diabetes onset (36). These findings suggest that the inhibitory effect of Tofacitinib on the IL-15 signaling pathway may be relevant to the pathogenesis of human type 1 diabetes.

### 3.3 Ruxolitinib

Ruxolitinib is a small-molecule JAK inhibitor that selectively inhibits JAK1 and JAK2. It is primarily used for the treatment of intermediate- or high-risk myelofibrosis (37), certain forms of drug-resistant polycythemia vera, and graft-versus-host disease (38).

In *in vitro* cell models, Ruxolitinib has been demonstrated to inhibit IFN-α-induced expression of HLA-I, CXCL10, MX1, and CHOP, as well as CXCL10 secretion in a dose-dependent manner (39). Additionally, it has been shown to block IFN-α/IFN-γ-mediated phosphorylation of STAT1/STAT2, ISRE/GAS reporter gene activity, and the expression of ER stress markers CHOP and XBP1 spliced isoform, while leaving ATF3 levels unaffected (26).

Experimental studies of the JAK1/JAK2 inhibitor ruxolitinib in the LEW.1WR1 rat model demonstrate that initiating administration prior to the onset of insulitis and maintaining treatment throughout the disease progression completely prevents diabetes development (40). Pancreatic tissues from rats treated with this agent for two weeks exhibited no histopathological evidence of insulitis or inflammatory cell infiltration. Although no insulitis was evident in any islets, a slight reduction in insulin staining was observed in all animals. These findings indicate that JAK pathway inhibition effectively blocks inflammatory cascades and preserves pancreatic islet function (40).

Case reports indicate that heterozygous STAT1 gain-of-function (GOF) mutations are associated with T1D and other autoimmune diseases (4, 41, 42). Ruxolitinib treatment in a STAT1 GOF patient with T1D and concomitant autoimmune manifestations resulted in reduced insulin dependence, with eventual discontinuation of insulin therapy (4). This clinical improvement correlated with restored NK cell function, evidenced by normalized perforin expression in CD56dim NK subsets and enhanced cytotoxic activity (42). These remarkable clinical outcomes in people with STAT1 gain-of-function mutations treated with ruxolitinib suggest that JAK inhibitors may reverse T1D in the presence of STAT1 gene mutations.

### 3.4 Deucravacitinib/BMS-986165

Deucravacitinib is a highly selective oral allosteric inhibitor that exerts its therapeutic effects by targeting the TYK2 pseudokinase (JH2) domain, a mechanism critical for treating immune-mediated diseases such as psoriasis and lupus nephritis. It has been recently approved for the treatment of plaque psoriasis (43–45).The drug binds to the ATP-binding site of the JH2 pseudokinase domain, stabilizing JH2’s autoinhibitory conformation over JH1 and thereby blocking its kinase activity and downstream pathological signaling (43, 46).

As a type IV allosteric inhibitor targeting non-catalytic sites (47), deucravacitinib uniquely binds to the TYK2 JH2 pseudokinase domain rather than competing directly with ATP in the JH1 catalytic pocket. This distinct mechanism enables selective TYK2 inhibition while sparing JAK1-3 activity, differentiating it from other JAK inhibitors like ruxolitinib and baricitinib (26, 43, 46). Preclinical studies have shown that under two distinct cytokine combinations—IFN-α + IL-1β (mimicking early insulitis) and IFN-γ + IL-1β (mimicking late insulitis)—deucravacitinib, baricitinib, and ruxolitinib all suppress IFN-α-induced STAT1/2 phosphorylation in pancreatic β-cells. However, deucravacitinib exhibits superior potency in blocking downstream ISRE-driven transcription and reducing pro-inflammatory gene expression, including HLA-I, MX1, and CXCL10 (12, 26). Importantly, unlike pan-JAK inhibitors, deucravacitinib preserves IFN-γ signaling due to its TYK2-specific allosteric inhibition. This selective mechanism protects β-cells from cytokine-induced apoptosis while maintaining insulin secretion and cellular viability (26). The specificity toward the TYK2 pseudokinase domain may offer a favorable safety profile, underscoring the therapeutic potential of pseudokinase-targeted strategies in type 1 diabetes and other autoimmune conditions.

*In vivo* studies using two murine models of T1D—RIP-LCMV-GP mice and NOD mice—demonstrated that TYK2 inhibition via deucravacitinib administration attenuated systemic and tissue-localized inflammation, preserved beta cell viability, and delayed autoimmune diabetes onset. Transcriptional profiling of pancreatic islets, pancreatic lymph nodes, and spleen during early disease progression revealed that TYK2 inhibitor modulated pathways linked to inflammation, stress signaling, secretory function, and immune activation. Furthermore, TYK2 inhibitor treatment altered innate and adaptive immune cell dynamics in blood and target tissues, suppressing the expansion of pathogenic T-BET+ cytotoxic T lymphocytes and fostering an immune milieu less conducive to beta cell destruction (48, 49).

### 3.5 Other JAK inhibitors

Current evidence supports the potential of JAK inhibitors with the sufix ‘-itinib’, such as tofacitinib and baricitinib, in treating T1D, and emerging research indicates that non-”-itinib” JAK inhibitors may also hold promise for T1D therapy.Therefore, we include these non-”-itinib” JAK inhibitors in our discussion to further explore the potential of JAK inhibitors in T1D treatment from a comprehensive perspective.

#### 3.5.1 Liraglutide

Liraglutide is a glucagon-like peptide-1 receptor agonist that also modulates the JAK/STAT pathway and therefore may show therapeutic potential in T1D. Liraglutide suppresses the JAK/STAT4 signaling pathway in Jurkat E6-1 T lymphocytes under hyperglycemic conditions, thereby inhibiting the production and secretion of IFN-γ (50). This mechanism demonstrates its immunomodulatory potential in attenuating autoimmune responses associated with T1D, suggesting therapeutic relevance for T1D management (50). In addition, a combination of anti-IL-21 therapy and liraglutide seems to preserve pancreatic beta-cells function in people with NOD mouse models and newly diagnosed T1D (51, 52).

Although adjunctive liraglutide therapy has been explored in T1D, it is crucial to recognize the potential for increased incidence of both symptomatic hypoglycemia and hyperglycemia with ketosis (53, 54). Therefore, close clinical monitoring is essential to optimize the risk-benefit balance and ensure patient safety.

#### 3.5.2 TYK2iA and TYK2iB

Other small-molecule inhibitors specifically targeting TYK2 may be advantageous for preventing or managing T1D in people carrying particular genetic variants. Two inhibitors, TYK2iA and TYK2iB, developed by Nimbus Lakshmi, have been tested in human insulin-producing EndoC-βH1 beta-cell line and dispersed human islets. They effectively prevent IFN-α-induced overexpression of HLA- class I, inflammatory chemokine production, and beta-cell apoptosis, without compromising human beta-cell function or increasing susceptibility to potential diabetogenic viruses (55).

#### 3.5.3 AZD1480

The JAK1/JAK2 inhibitor AZD1480 mitigates cytokine-induced beta-cell damage in both murine and human models by suppressing MHC class I upregulation (16). This intervention disrupts direct CD8^+^ T cell-beta cell interactions and attenuates immune cell infiltration into pancreatic islets (16). In NOD mouse models, AZD1480 treatment prevents autoimmune diabetes onset and reverses disease progression in newly diagnosed cases, thereby establishing a mechanistic rationale for repurposing clinically approved JAK inhibitors in T1D therapy (16).

#### 3.5.4 ABT 317

In NOD mice, twice-daily ABT-317 (a JAK1-selective inhibitor) for 40 days reversed diabetes in 94% of new-onset cases, with 44% sustaining normoglycemia 60 days post-treatment. ABT-317 exhibits dual inhibitory effects: it suppresses IFN-γ signaling while concurrently inhibiting common γ-chain cytokine signaling (17). Notably, common γ-chain cytokines predominantly signal through the JAK/STAT pathway. This dual mechanism prevents IFN-γ-induced MHC class I overexpression on beta-cells and inhibits the proliferation of effector memory CD8+ T cells (17). Simultaneously, by blocking γ-chain cytokine signaling, ABT-317 further reduces beta-cell vulnerability to CD8+ T cell recognition. This dual targeting outperforms isolated IFN-γ blockade, offering a novel strategy for T1D remission (17).

#### 3.5.5 Tyrphostin AG490

Tyrphostin AG490 provides durable immunomodulatory effects in restoring glucose metabolism and may be an effective and safe agent for preventing the onset of clinical diabetes. It inhibits tyrosine phosphorylation in the JAK/STAT pathway, blocking activation of both JAK and STAT family members (56). Notably, AG490 is 4.3 times more potent against JAK2 compared with JAK3 (56). Additionally, AG490 inhibits IL-2-induced T cell proliferation by suppressing JAK3 in a dose-dependent manner in the D10 and CTLL-2 T cell lines (57). It also impedes *in vivo* differentiation of antigen-specific Th1 cells (58), and has been proven effective in preventing allograft rejection (59, 60)experimental autoimmune diseases (57), as well as in cancer therapy (61, 62).

A study treated female NOD mice with AG490 (1 mg/mouse) and found that starting treatment at 4 or 8 weeks of age significantly lowered blood glucose levels, preventing the development of autoimmune diabetes. Furthermore, most recovered mice maintained normal glycemic control for up to 30 weeks after discontinuation of AG490 (15). These findings suggest AG490’s potential as a promising therapeutic candidate for preventing or reversing T1D.

#### 3.5.6 LN3103801

The JAK1/JAK2 inhibitor LN3103801 prevents/reverses hyperglycemia by dual blockade of IFN-γ-mediated MHC-I overexpression in beta-cells and γc cytokine-driven T-cell activation, without compromising antitumor efficacy in NOD mice (63). This study provides preclinical validation for repurposing JAK1/JAK2 inhibitors to mitigate checkpoint inhibitor-associated diabetes while preserving anticancer immunity.

#### 3.5.7 BMS-986202

BMS-986202 is a clinical-stage TYK2 inhibitor that selectively binds to the JH2 pseudokinase domain of TYK2, allosterically inhibiting its kinase activity and downstream JAK/STAT signaling pathways (64).

*In vivo*, BMS-986202 delayed diabetes onset in both the NOD mouse model and the RIP-LCMV-GP model—a model designed to express the lymphocytic choriomeningitis virus glycoprotein under the rat insulin promoter—by attenuating systemic and pancreatic inflammation and preserving beta-cell mass (49).

### 3.6 Herbal ingredients

Traditional herbal constituents widely used in Asian traditional medicine, such as punicalagin and asaronic acid, have demonstrated potential in improving peripheral insulin sensitivity. Preclinical and animal model studies indicate that this effect may be mediated through JAK/STAT pathway modulation (65). Furthermore, these compounds exhibit immunomodulatory properties by directly or indirectly regulating the JAK/STAT signaling cascade, suggesting their ability to mitigate T1D progression via coordinated metabolic improvement and immune regulation (65).

#### 3.6.1 Punicalagin

Punicalagin is a naturally occurring polyphenol from pomegranate. It modulates immune responses (66) and is beneficial for both chronic (67) and acute inflammatory conditions (68) Its mechanisms of action include regulating the NF-κB, mitogen-activated protein kinase (MAPK), IL-6/JAK/STAT3, and the phosphoinositide 3-kinase (PI3K)/protein kinase B (Akt)/mechanistic target of rapamycin (mTOR)signaling pathways (69). Studies using STZ-induced T1D rat models (70) and RINm5F cells (71) have shown that punicalagin reduces inflammation and oxidative stress, preventing pancreatic damage and protecting beta-cells. Thus, it holds potential for preventing and treating diabetes and its complications (65).

#### 3.6.2 Asaronic acid

Asaronic acid is a natural phenylpropanoid compound extracted from the traditional Chinese medicinal herb Asarum spp. (known as Xi Xin) with anti-inflammatory, analgesic, and neuroprotective properties. It is widely used to alleviate pain and inflammation-related diseases.*In vitro* experiment proves that it restricts the diabetic macrophage activation toward the M1 phenotype by inhibiting Toll-like receptor 4 (TLR4)-/IL-6–induced NF-κB/JAK2-STAT1/2 signaling and modifying the interaction of advanced glycation end products with their receptors in glucose-stimulated macrophages. Consequently, it may offer potential therapeutic effects against diabetes-related complications (72).

## 4 Potential use of JAK inhibitors in preventing or treating CPI-DM

Immune checkpoint inhibitors are increasingly used in treating various advanced malignancies (73).Moreover, attention to endocrine dysfunctions—such as immune checkpoint inhibitor–induced diabetes—is also growing (74, 75). CPI-DM typically arises in association with PD-1 inhibitors and is clinically considered as a “T1D-like” disease, although with different triggers. A systematic review and meta-analysis reported that CPI-DM, often developing within three months of initial PD-1/PD-L1 inhibitor exposure, typically presents with acute diabetic complications such as diabetic ketoacidosis, characterized by rapid onset and severe hyperglycemia, with a higher rate of diabetic ketoacidosis observed in individuals testing positive for T1D-associated autoantibodies (74). Although the precise mechanisms by which these drugs perturb endocrine homeostasis remain unclear, their clinical phenotype closely resembles that of T1D—marked by immune dysregulation and an almost complete loss of pancreatic beta-cell function (76).

Recent studies suggest that immune checkpoint inhibitor resistance may be associated with defects in antigen presentation due to mutations in the JAK/STAT pathway (77–79). Moreover, the IFN signaling pathway, involving JAK1/2–STAT1/2/3–IRF1, has been shown to regulate antigen presentation and PD-L1 expression (80). Based on this background, here, we will summarize the available findings about CPI-DM and JAK inhibitors.

Published data suggest that JAK inhibitors may have beneficial effects in regulating CPI-DM. In NOD mice with anti–PD-L1–induced hyperglycemia, administration of a JAK1/JAK2 inhibitor resulted in the reversal of diabetes (63). This effect may involve JAK1/JAK2 inhibitors blocking IFN-γ-driven upregulation of MHC-class I on beta-cells and suppression of T-cell proliferation mediated by cytokines that utilize the common γ-chain receptor (63). Studies have demonstrated that PD-L1 expression is elevated in islets from donors with T1D compared to healthy controls (81). Subsequent *in vitro* experiments using human EndoC-βH1 cells or dispersed human islets revealed that IFN-α and IFN-γ upregulate PD-L1 expression via IRF1 induction. Importantly, this IFN-driven PD-L1 upregulation was inhibited by the JAK1/2 inhibitor ruxolitinib (81). Furthermore, elevated levels of phosphorylated STAT1/3 in T1D patients correlate with higher expression of inhibitory molecules such as PD-L1, PD-L2, and PD-1, which impact immune regulatory mechanisms (82). By suppressing STAT phosphorylation, JAK inhibitors may mimic the effects of PD-1/PD-L1 inhibitors. Nonetheless, further basic research and clinical trials are needed.

## 5 Common adverse effects of JAK inhibitors in clinical treatment

Recent studies suggest that JAK inhibitors may be associated with hypoglycemia. Initially, no hypoglycemia warnings were included in the EU or FDA product information for Baricitinib, Tofacitinib, Upadacitinib, or Filgotinib. However, a 2022 report from the European Medicines Agency’s pharmacovigilance database analyzed 43 cases in which these JAK inhibitors were co-administered with antidiabetic drugs. Eight patients showed improvements in hypoglycemic symptoms after discontinuing or reducing the JAK inhibitor or adjusting the dosage of antidiabetic agents. Additionally, both rapid-acting and long-acting insulin doses were reduced by 30%. These findings indicate that JAK inhibitors may induce hypoglycemia while lowering blood glucose (83).

Two studies found that adding liraglutide to insulin therapy in patients with T1D might increase the risk of symptomatic hypoglycemia and ketotic hyperglycemia, which may limit its clinical use (54, 84), Given the unclear benefits and risks of JAK pathway inhibitors in T1D, clinicians should exercise caution regarding insulin dosage adjustments and remain vigilant for potential hypoglycemia.

In people with active rheumatoid arthritis and other inflammatory rheumatic diseases, mortality and serious adverse events associated with JAK inhibitors primarily involve infections, atherosclerotic cardiovascular disease, and malignancies (85–88). Notably, some of them showed dyslipidemia, yet their atherosclerosis index was generally normal (88, 89). Regarding infections and malignancies—particularly non-melanoma skin cancer and other malignancies—the associated risk appeared similar to that of the general population (90, 91).

In the BANDIT study, safety outcomes between the baricitinib and placebo groups showed comparable frequencies and severity of adverse events, with median adverse events per patient being 2 vs. 3 (P=0.14). Seven serious adverse events occurred, including ketoacidosis in both groups (2 in baricitinib, 1 in placebo), though none were attributed to baricitinib. Infections such as upper respiratory and skin infections occurred at similar rates, with one herpes zoster case in the baricitinib group. No clinically significant differences in lipid profiles, liver function, or creatinine levels were observed. Notably, transient insulin cessation during COVID-19 illness did not significantly alter glycemic control or C-peptide levels at follow-up (5).

According to existing registration trials and publicly available data, the risk of major adverse cardiovascular events, venous thromboembolism, malignancies, and infections (excluding herpes zoster) is similar between JAK inhibitors and disease-modifying anti-rheumatic drugs. Mortality and the incidence of serious adverse events are also comparable with those of the general population (89, 92, 93).

Mild neutropenia and lymphopenia occur at frequencies similar to placebo, although severe lymphopenia—if present—can be associated with serious infection (85, 87, 94). Common serious infections include pneumonia, herpes zoster, and urinary tract infections. However, there is no evidence of a marked rise in other severe infections (85, 89, 95). In addition, a small percentage of patients starting baricitinib may experience declines in hemoglobin and reticulocyte counts, although fewer than 1% progress to clinically significant anemia (85, 88, 96). Notably, while herpes zoster was reported in baricitinib trials, broader data align with the observation that infection risks (excluding herpes zoster) remain comparable to conventional therapies (5).

## 6 Future perspectives

Leveraging preclinical evidence that JAK inhibition preserves beta-cell function, researchers are now testing whether these agents can alter the trajectory of T1D by addressing its root cause: the immune system’s attack on insulin-producing cells.

Since March 2025, the TrialNet JAKPOT T1D Study has been underway as a multi-regional clinical trial conducted across Europe, Australia, and North America. This trial aims to evaluate the efficacy of two JAK inhibitors—abrocitinib(an FDA-approved treatment for atopic dermatitis) and ritlecitinib(an investigational drug currently being studied for alopecia areata and Crohn’s disease)—in preserving endogenous insulin production in individuals aged 12–35 years with recent-onset T1D. Eligible participants must have been diagnosed with T1D within the preceding three months. This randomized, double-blind, placebo-controlled trial will enroll 78 participants, who will be assigned equally to receive abrocitinib, ritlecitinib, or a placebo. Researchers hypothesize that JAK inhibitors could reduce immune-mediated destruction of pancreatic beta-cells, potentially preserving residual insulin secretion. If confirmed, this effect might improve long-term glycemic control and lower the risk of diabetes-related complications. Success in this trial could pave the way for future studies exploring these therapies in earlier pre-symptomatic stages of T1D to delay or prevent clinical disease onset (https://www.trialnet.org/our-research/newly-diagnosed-t1d/jakpot-t1d).

Looking beyond current trials, future research should prioritize enhancing the selectivity and safety profiles of JAK inhibitors, particularly by targeting specific JAK isoforms. For instance, the allosteric inhibition of TYK2, as demonstrated by deucravacitinib, offers a promising therapeutic avenue by circumventing the broader immunosuppressive effects associated with pan-JAK inhibition. The development of novel molecules that exploit pseudokinase domain binding, or technologies like proteolysis-targeting chimeras (PROTACs), may enable more refined modulation of JAK-STAT signaling with fewer adverse events.

Although current data on combination therapy are limited, future research may explore the potential synergy between JAK inhibitors and other immunomodulatory approaches, such as anti-IL-21 therapy or antigen-specific tolerance strategies. While combination strategies remain exploratory, the continued advancement of JAK inhibitors in clinical research underscores the importance of robust long-term evaluation. As these agents advance in development, long-term follow-up studies will be crucial to evaluate their impact on beta-cell preservation, infection risk, cancer surveillance, and overall metabolic outcomes in pediatric and adolescent populations. Integrating biomarker-driven endpoints in future trials may help bridge the gap between mechanistic insights and clinical application, bringing us closer to disease-modifying therapies—and potentially prevention—of T1D.

## 7 Conclusion

This review further discuss the pathogenic role of the JAK/STAT pathway in T1D and highlights research advances on key therapeutic agents. Notably, combining JAK inhibitors with immune checkpoint inhibitors may reduce the incidence of CPI-DM. However, possible adverse drug reaction should be considered. Further studies are essential to validate these findings and inform clinical practice.
